# COVID-19 Vaccine Hesitancy among the Younger Generation in Japan

**DOI:** 10.3390/ijerph182111702

**Published:** 2021-11-07

**Authors:** Mostafa Saidur Rahim Khan, Somtip Watanapongvanich, Yoshihiko Kadoya

**Affiliations:** School of Economics, Hiroshima University, Hiroshima 739-8525, Japan; somtip.w@gmail.com (S.W.); ykadoya@hiroshima-u.ac.jp (Y.K.)

**Keywords:** COVID-19 pandemic, vaccine hesitancy, younger generation, gender, age, regression, Japan

## Abstract

Japan has vaccinated its older population; a mass vaccination program for younger citizens is underway. Accordingly, this study investigated vaccine hesitancy among younger Japanese citizens. We used online panel survey data from the Hiroshima Institute of Health Economics Research of Hiroshima University and applied probit regression models. Our study found that vaccine hesitancy among younger people was significantly higher than among older people. Moreover, vaccine hesitancy was significantly higher among younger women than younger men and inter-age-group differences in vaccine hesitancy were higher for younger men than for younger women. Regression demonstrated that subjective health status and anxiety about the future were significantly associated with vaccine hesitancy among younger women and younger men of all ages, respectively. Furthermore, marital status, university degree, anxiety about the future, and myopic view of the future had specific associations with vaccine hesitancy among younger women of different ages, while subjective health status, university degree, having children, financial literacy, household income and assets, and myopic view about the future had specific associations with vaccine hesitancy among younger men of varying ages. Therefore, these results suggest that policymakers should consider the diversity among the younger generation while developing effective, tailored communication strategies to reduce their vaccine hesitancy.

## 1. Introduction

The impact and consequences of COVID-19 vaccines globally have been revealed gradually. COVID-19 infections and serious illnesses have significantly reduced in places with high vaccination rates [1]. Many social and economic activities have returned to normal. However, the ultimate success of the vaccination program depends on how quickly a significant group of people is vaccinated so that the spread of viral infection is effectively contained. Japan launched its vaccination program in February 2021 and successfully vaccinated priority groups such as older people and healthcare workers. To effectively control the spread of viral infection as soon as possible, mass vaccination for adults has been in progress in Japan since July, 2021. As of October 1, 2021, 89% of people older than 65 years have been fully vaccinated; moreover, 64% of the entire population has received at least one dose of the vaccine [2]. As the target for the older population has been achieved, the government has focused on younger adults and the mass vaccination program. Concern about vaccine hesitancy among younger people is increasing because of lessons learned from other countries [3]. Many countries have experienced higher vaccine hesitancy among younger generations, slowing the progress of vaccination programs [4,5,6,7,8]. Moreover, in many countries, vaccine hesitancy has been found to be higher among younger women [9,10,11,12]. Global evidence of intergenerational and intergender differences in vaccine hesitancy makes it necessary to closely examine the vaccine status in Japan. However, few studies in Japan have comprehensively investigated vaccine hesitancy among the younger generation. To bridge this gap, we investigated vaccine hesitancy among younger Japanese populations across various gender- and age-based strata. Moreover, we estimated the influence of the younger generation’s socio-economic orientation on vaccine hesitancy. Findings will help policymakers develop specific outreach strategies for the younger generation and create motivating messages to reduce vaccine hesitancy. Framing an effective communication strategy tailored to the younger generation is necessary, as a one-size-fits-all communication approach may not be effective for groups sharing different values and priorities [13,14].

As COVID-19 vaccines have been approved on an emergency basis within a short period of time, concerns persist within the general population about vaccine safety and efficacy. Vaccines have not been available long enough to remove negative perceptions. Vaccine hesitancy is defined as delayed acceptance, reluctance, or refusal of vaccines despite their availability [15,16]. Thus, vaccine-hesitant people may refuse vaccines, delay vaccines, accept some vaccines but refuse others, or accept vaccines but remain unsure while doing so. The World Health Organization reported that the causes of vaccine hesitancy are complex and vary across time, place, and vaccine types [15]. There are many global examples of vaccine hesitancy, even when there is improved and easy access to vaccines, including Human Papillomavirus vaccine hesitancy in Japan and measles vaccine hesitancy in Europe [15]. Vaccine hesitancy has individual behavioral and national cultural aspects as well. Historically, Japan is a country with lower vaccine confidence and higher hesitancy [17,18]. A closer investigation of COVID-19 vaccine hesitancy is needed to determine which younger socio-demographic groups are more likely to be vaccine-hesitant in a traditionally vaccine-hesitant country such as Japan.

Globally, there have been a large number of studies within a limited time on COVID-19 vaccine hesitancy among younger adults [4,5,9,10,19,20], including some with evidence of vaccine hesitancy among younger people in Japan [21,22,23,24]. Although these studies demonstrated higher vaccine hesitancy among younger adults in Japan, the magnitudes of hesitancy were somewhat different, possibly due to differences in the timing of data collection and definitions of vaccine hesitancy. Results of ongoing medical experiments on vaccine safety, efficacy, and modes of vaccine administration have continued to mold attitudes about vaccination. Yoda and Katsuyama [22] and Machida et al. [23] found the hesitancy rate in Japan during the trial phase of the vaccination program to be 34.3% and 37.9%, respectively. Kadoya et al. [21] found the rate to be 53%; Okubo et al. [24] found the rate to be 11% after the rollout of the vaccination program. Despite differences in vaccine hesitancy at different stages of the vaccination program, previous studies have provided consistent evidence related to gender and age associations with vaccine hesitancy. Kadoya et al. [21] reported that women were more vaccine-hesitant than men, while middle-aged populations were less vaccine-hesitant than their younger and older counterparts. Kadoya et al. [21] divided their study’s full sample into older and younger subsamples as well as subsamples of men and women and found that socio-economic factors associated with vaccine willingness differed for gender- and age-based subsamples. Okubo et al. [24] found that vaccine hesitancy was much higher among younger people, particularly among younger women. Similarly, Yoda and Katsuyama [22] and Machida et al. [23] found higher vaccine hesitancy among younger respondents and women. Although evidence of higher vaccine hesitancy among younger people and women is evident, neither the Yoda and Katsuyama nor the Machida et al. study investigated socio-economic covariates of vaccine hesitancy across gender-and age-based subsamples [22,23]. A comprehensive study on vaccine hesitancy in Japan among gender- and age-based subsamples is needed, as data from other countries show variance among younger adults in terms of vaccine hesitancy and the reasons associated with hesitancy [5,9,10,19].

## 2. Material and Methods

### 2.1. Data

This study used the panel data from the Household Behavioral and Financial Survey by Hiroshima Institute of Health Economics Research of Hiroshima University [25]. The research was conducted by Nikkei Research, a prominent research company in Japan, whose database is representative of the Japanese population and is considered one of the largest research databases in the country. The first online survey was conducted from February 20 to 25, 2020, at the beginning of the COVID-19 pandemic. The second online survey was conducted after a year from February 19 to 26, 2021, at the commencement of the COVID-19 vaccination program in Japan. The datasets contain socio-economic characteristics and preferences of the Japanese population aged 20 and older; the total number of observations within the surveys are 17,463 and 6103 in 2020 and 2021, respectively.

This study used the 2021 dataset, containing questions about acceptance of the COVID-19 vaccines and respondents’ socio-economic status [25]. For some demographic characteristics and preferences, which were unavailable from the 2021 survey, including age, gender, place of residence, education, number of children, and financial literacy, the 2020 dataset was used instead. The final sample contained 4253 observations, approximately 70% of the total observations in the 2021 dataset. Observations with missing data on important socio-economic variables, such as household assets and household income, were excluded from the sample (1850 observations).

### 2.2. Variable Definitions

The dependent variable “vaccine hesitancy” was assessed using question: “Once the COVID-19 vaccination becomes available free of charge, I will take it soon.” It was responded using a 5-point rating scale as follows:

1 = “Strongly disagree”;
2 = “Disagree”;
3 = “Neither agree nor disagree”;
4 = “Agree”;
5 = “Strongly agree”.

We considered “vaccine hesitancy” a binary variable, where 1 indicated answers 1, 2, or 3 to the above question, following Fisher et al. [26], and 0 indicated answers of 4 or 5.

Gender, age, marital status, number of children, living condition, place of residence, education, employment status, household assets, and household income were included as demographic and socio-economic variables. Financial literacy was included in the model as a proxy for rational decision-making ability in health-related behaviors, as suggested by previous studies [27,28,29]. Subjective health status, anxiety about the future, myopic view of the future, and risk preference were also included in the model specifications. Table 1 defines all variables.

### 2.3. Statistical Analysis

Descriptive statistics were utilized to show the distribution of respondents’ vaccine hesitancy along with their demographic, socio-economic, and psychological backgrounds. Vaccine hesitancy stratified by gender and age were determined, together with test statistics and significance levels. Finally, the association between socio-economic factors and vaccine hesitancy were explored in Equation (1).

(1) $$Y_{i1}=f\left(X_{i},\varepsilon_{i}\right)$$

$Y_{1}$ is vaccine hesitancy, X is a vector of individual characteristics, and ε is the error term. We used probit regression to estimate Equation (2), where the dependent variable was a binary variable.

We conducted correlation and multicollinearity tests for all models (available upon request). The results showed a weak relationship between explanatory variables (lower than 0.70) and revealed no significant multicollinearity in any model.

The full model specification of Equation (1) is as follows:

(2) $$\begin{array}{l} \mathrm{Vaccine}\;{\mathrm{hesitancy}}_{i}\\ \;=\beta_{0}+\beta_{1}{\mathrm{male}}_{i}+\beta_{2}{\mathrm{age}}_{i}+\beta_{3}{\mathrm{age}\;\mathrm{squared}}_{i}\\ \;+\beta_{4}\mathrm{spouse}+\beta_{5}{\mathrm{children}}_{i}+\beta_{6}{\mathrm{living}\;\mathrm{alone}}_{i}\\ \;+\beta_{7}{\mathrm{living}\;\mathrm{in}\;\mathrm{central}\;\mathrm{area}}_{i}+\beta_{8}{\mathrm{university}\;\mathrm{degree}}_{i}\\ \;+\beta_{9}{\mathrm{employed}}_{i}+\beta_{10}{\log\;\mathrm{of}\;\mathrm{household}\;\mathrm{income}}_{i}\\ \;+\beta_{11}{\log\;\mathrm{of}\;\mathrm{household}\;\mathrm{assets}}_{i}+\beta_{12}{\mathrm{financial}\;\mathrm{literacy}}_{i}\\ \;+\beta_{13}{\mathrm{subjective}\;\mathrm{health}\;\mathrm{status}}_{i}\\ \;+\beta_{14}{\mathrm{anxiety}\;\mathrm{about}\;\mathrm{the}\;\mathrm{future}}_{i}\\ \;+\beta_{15}{\mathrm{myopic}\;\mathrm{view}\;\mathrm{of}\;\mathrm{the}\;\mathrm{future}}_{i}\\ \;+\beta_{16}{\mathrm{level}\;\mathrm{of}\;\mathrm{risk}\;\mathrm{preference}}_{i} \end{array}$$

## 3. Results

### 3.1. Descriptive Statistics

Table 2 shows descriptive statistics of the main variables. Results show that 53% of respondents were hesitant to take the vaccine. For demographic variables, 65% of the respondents were men; average age was 50.32 years. For household status, 66% of the respondents had spouses, 57% had children, 20% lived alone, and 61% lived in Japan’s central area (around Tokyo and Osaka metropolises). Approximately 62% of the respondents held a university degree; 64% were currently employed. Respondents’ average annual household income and household assets were JPY 6.34 million and JPY 19.80 million, respectively. The average financial literacy score of respondents was 0.65. On average, respondents rated their subjective health, anxiety about the future, and myopic view of the future at 3.24, 3.71, and 2.69 out of 5, respectively. Respondents had an average risk preference of 46%, indicating slight risk aversion.

The full sample was classified into several age- and gender-based subsamples. Additional descriptions of vaccine hesitancy are provided in Table 3, which shows that vaccine hesitancy rates were higher in women than in men. Moreover, subsamples of younger men and women were more hesitant than older people. Overall results show a difference in vaccine hesitancy between men and women and between younger and older subsamples. However, differences in vaccine hesitancy among various younger subgroups were lower in subsamples of women than in subsamples of men.

### 3.2. Regression Results

The results of probit regression analyses of vaccine hesitancy of men and women of different age groups are described below. The entire sample was divided into younger (<65 years) and older (≥65 years) subsamples for both women and men; regression results were reported. Next, the younger men and women subsamples were divided into three age groups: <35, 35–49, and 50–64 years; regression results were reported for each group.

Table 4 presents the regression results for vaccine hesitancy among younger and older women. Results showed that age, age squared, having children, and subjective health status were significantly associated with vaccine hesitancy among younger women, while age, age squared, and log of household income were significantly associated with vaccine hesitancy among older women. These results indicate that age influenced vaccine hesitancy among both younger and older women. However, having children and subjective health status had additional influence on younger women’s vaccine hesitancy.

Table 5 shows regression results for vaccine hesitancy among younger women after dividing the women into three age groups. Results show that having a spouse, university degree, subjective health status, and anxiety about the future were significantly associated with vaccine hesitancy among the youngest subsamples, while age, age squared, having children, and subjective health status were significantly associated with vaccine hesitancy among the 35–49 age group; only subjective health status was significantly associated with vaccine hesitancy among the 50–64 age group. These results indicate that subjective health status was the only factor that significantly influenced vaccine hesitancy among all younger women in the sample. Moreover, having a spouse, university degree, and anxiety about the future had additional influence on vaccine hesitancy among the youngest subsamples, while age structure and having children had additional influence on vaccine hesitancy among the 35–49 age group.

Table 6 shows regression results for vaccine hesitancy among younger and older men. The results show that age, age squared, log of household income, log of household assets, financial literacy, subjective health status, anxiety about the future, and myopic view about the future were significantly associated with vaccine hesitancy among younger men, while having children, log of household income, and log of household assets were significantly associated with vaccine hesitancy among older men. These results indicate that log of household income and log of household assets influenced vaccine hesitancy among both younger and older men. However, age structure, financial literacy, subjective health status, anxiety about the future, and myopic view about the future had additional influence on younger men’s vaccine hesitancy.

Table 7 shows regression results for vaccine hesitancy among younger men after dividing the men into three age groups. Results show that university degree, log of household assets, financial literacy, and anxiety about the future were significantly associated with vaccine hesitancy among the youngest subsamples while log of household income, financial literacy, subjective health status, anxiety about the future and the level of risk preference were significantly associated with vaccine hesitancy among the 35–49 age group. Having children, log of household income, subjective health status, anxiety about the future, and myopic view about future were significantly associated with vaccine hesitancy among the 50–64 age group. These results indicate that anxiety about the future was the only factor that significantly influenced vaccine hesitancy among all younger men. Moreover, financial literacy influenced vaccine hesitancy among the youngest men and the 35–49 age group, while log of household income and subjective health status influenced vaccine hesitancy among the 35–49 and 50–64 age groups. Finally, university degree and log of household assets influenced vaccine hesitancy among the youngest men, while the level of risk preference influenced vaccine hesitancy among the 35–49 age group and having children and myopic view about future influenced vaccine hesitancy among the 50–64 age group.

### 3.3. Robustness Check

To check the robustness of the results, we performed multinomial probit regression with vaccine non-hesitancy among younger generation as the base category. Table 8 shows the coefficients of the multinomial probit regression model for women.

The results showed that subjective health status was significantly associated with vaccine hesitancy among all younger women in the sample. Moreover, age structure, having a spouse, university degree, and anxiety about the future additionally influenced vaccine hesitancy among the youngest subsample, while age structure and having children influenced vaccine hesitancy among the 35–49 and 50–64 age groups. Moreover, household income was significantly associated with vaccine hesitancy among the 50–64 age group. 

Table 9 shows the coefficients of the multinomial probit regression model for men. The results showed that anxiety about the future is significantly associated with vaccine hesitancy among all younger men; age structure, university degree, and financial literacy influenced vaccine hesitancy among the youngest men; age, financial literacy, and subjective health status influenced vaccine hesitancy among the 35–49 age group; and age structure, having children, household income, subjective health status, and a myopic view about the future influenced vaccine hesitancy among the 50–64 age group. Overall, the results of the multinomial probit regression models were consistent with our original findings that subjective health status and anxiety about the future are significantly associated with vaccine hesitancy among all younger women and younger men, respectively. Moreover, other age-specific covariates of vaccine hesitancy were found for both women and men.

We also conducted weighted probit regression models to ensure proper sample representation within each group (results are not shown in the manuscript to save space but are available upon request). The weighted probit regression coefficients also showed that subjective health status is significantly associated with vaccine hesitancy among all younger women, and anxiety about the future is significantly associated with vaccine hesitancy among all younger men. Moreover, having children, university degree, employment status, and household income was specifically associated with vaccine hesitancy among younger women of different ages, while having children, household income and assets, financial literacy, subjective health status, and a myopic view about the future was specifically associated with vaccine hesitancy among younger men of varying ages.

## 4. Discussion

The ongoing mass vaccination program targeting younger adults in Japan has passed into an important phase. The success of the mass vaccination program depends on the willingness of the younger generation to participate in the program. However, known global vaccine hesitancy among younger people is an impediment to achieving targeted herd immunity [4,5,6,7,8]. Few studies have documented the prevalence of higher vaccine hesitancy among younger people and women in Japan [21,22,23,24]. As vaccine hesitancy is prevalent among younger generations and women, and as there is no comprehensive study in Japan investigating this issue further, this study investigated vaccine willingness and hesitancy among younger Japanese, classified by gender and age groups. Moreover, the associations of demographic, socio-economic, and psychological aspects and preferences related to vaccine willingness and hesitancy among younger generations were also investigated.

### 4.1. Prevalence of Vaccine Hesitancy among Younger People Stratified by Gender and Age

This study provided evidence of higher vaccine hesitancy among younger people compared to their older counterparts. Vaccine hesitancy was higher for younger women than for younger men. Inter-age group differences in vaccine hesitancy were lower for women, indicating that all younger women are more vaccine-hesitant than older women. However, younger men aged 21–49 years were more vaccine-hesitant than those in the 50–64 age group. Historically, Japan is a country with lower vaccine confidence and higher vaccine hesitancy [17,18]; therefore, these results are not surprising but rather provide specific evidence of how vaccine hesitancy varies by gender and age. The current study results are consistent with the findings of previous studies on COVID-19 vaccine acceptance in Japan [21,22,23,24] and other countries [5,9,10,19,20,26].

There have been several global studies attributing vaccine hesitancy among younger generations to lower risk perception of the disease, perceived side effects, trust in vaccine, media misinformation, and socio-economic issues [4,30,31,32,33]. As complications, hospitalization, and mortality from the COVID-19 viral infection are found to be less severe for younger populations [34,35], the need for vaccination may not be sufficiently emphasized. Vaccine hesitancy among younger women may be related to additional concerns about reproductive health and breastfeeding [11,36,37].

### 4.2. What Explains Vaccine Hesitancy among Younger Men and Women?

The uncontrolled observations presented in the descriptive statistics highlights three important scenarios of vaccine hesitancy in Japan. First, vaccine hesitancy was significantly higher among younger people compared to their older counterparts. Second, vaccine hesitancy was significantly higher among younger women than younger men. Third, inter-age-group differences in vaccine hesitancy were higher for younger men than for women. Overall, findings suggest that vaccine willingness and hesitancy should be studied by specific age- and gender-based subgroups to better understand factors associated with vaccine-acceptance decisions. Regression results show that there were only a few factors associated with vaccine hesitancy among younger men and women across all groups. However, there were factors associated with the vaccine hesitancy of younger men and women of specific age groups.

Subjective health status was the most consistent factor associated with vaccine hesitancy among all age groups of younger women. Findings indicate that younger women who were confident about their health were less vaccine-hesitant than their less confident counterparts. These findings are consistent with those of Soares et al. [7], who found higher vaccine willingness among people with better perceived health status. There could be two explanations for why the subjective health status of younger women is so important for vaccine-acceptance decisions. First, younger women who are confident about their health status may perceive vaccination as a way to remain healthy and safe from COVID. Since sound health status is the outcome of positive health behavior, younger women are likely to perceive vaccination as a rational decision. Second, the safety of the vaccine may also be relevant. Previous studies have provided evidence of concerns regarding vaccine safety and side effects among reproductive-age women [11,36,37]. Younger women with lower confidence in their health may be more concerned about possible side effects and may be hesitant to be vaccinated.

In addition to subjective health status, vaccine hesitancy among women in the youngest age group (younger than 35 years) were strongly associated with marital status, university degree, and anxiety about the future. Since women of this age group are more concerned about vaccine safety in terms of reproductive health and breastfeeding, findings must be explained considering these viewpoints as well. Overall, findings are consistent with rational vaccination decisions. Younger women with more education are likely to be cognizant of the benefits of vaccines and able to assess misinformation about vaccines, making them more willing and less hesitant to be vaccinated. Klugar et al. [38] and Khuc et al. [39] provided evidence that understanding the importance of vaccines, concern for the side effects, and lack of access to information are important determinants of vaccine hesitancy among younger adults. Health authorities in Japan have approved mRNA vaccines produced by Moderna and Pfizer, which are not associated with serious side effects. Since viral vector vaccines such as the AstraZeneca vaccine are rarely used in Japan, news of serious side effects related to the AstraZeneca vaccine has not been relevant for Japan [40]. Salerno et al. [41] found lower vaccine hesitancy among students under 30 years of age for mRNA vaccines than for viral vector vaccines. Several studies have confirmed that mRNA vaccines do not have proven adverse effects on pregnancy and breastfeeding [42,43]. An educated person is likely to understand vaccine safety and side effects, making it easier to make the correct vaccination decision. Findings of Soares et al. [6] on higher vaccine willingness among people with a university degree support this explanation.

Women are likely to accept vaccines as a way to reduce anxiety about the future, as global vaccination is seen as a path to restore normalcy in life. Soares et al. [7] found that people who have feelings of anxiety, agitation, and sadness are more likely to be vaccinated. Thus, women with less education and less anxiety about the future are likely to be vaccine-hesitant because they may not fully understand the benefits of the vaccine, place higher importance on negative propaganda about the vaccine, and be careless about the need for vaccine for their future.

Anxiety about the future is often associated with vaccine hesitancy decisions among younger men of all age groups. A factor associated with the youngest women’s vaccine hesitancy decisions, vaccination as a way to reduce anxiety about the future, appears to be a rational decision for younger men as well. The COVID-19 pandemic has changed economic and social lives so much that younger men, who comprise a significant percentage of the working population, are likely to be anxious not only about their health but also about economic conditions and social relationships. As the introduction of vaccines appears to be a gateway for normal life, younger men are likely to be vaccinated in order to return to their usual lives. On the other hand, younger people who are not anxious about the future are more vaccine-hesitant, likely because they are less concerned about the risk of future economic and social suffering due to the pandemic. Vaccination decision-making based on a rationality viewpoint is consistent with this study’s findings: that financial literacy and university degree were associated with vaccination decisions among the youngest men, subjective health status was associated with vaccination decisions among the 35–49 and 50–64 age groups, and a myopic view about the future was associated with vaccination decisions among the 50–64 age group.

## 5. Conclusions

There is global evidence on higher vaccine hesitancy among the younger generation, including in Japan. However, there is a lack of comprehensive Japanese literature to investigate this issue further. To bridge this gap, we investigated vaccine hesitancy among younger Japanese populations across various gender- and age-based strata. Our study shows that vaccine hesitancy is higher among younger men and women than among older people and that younger women are more vaccine-hesitant than younger men. Moreover, inter-age-group differences in vaccine hesitancy were higher among younger men than among younger women. Regression results demonstrated that subjective health status was the most influential factor for vaccine hesitancy among younger women of all age groups while anxiety about the future was the most influential factor for vaccine hesitancy among younger men of all age groups. However, some socio-economic and psychological factors are associated with vaccine hesitancy among younger men and women of specific age groups. Study findings suggest that communication strategies to reduce vaccine hesitancy should be tailored to younger populations of specific age groups rather than reliance on a one-size-fits-all communication strategy.

The study had certain limitations. First, as our study involved self-reported data, we cannot ignore the possibility of biases such as desirability bias or halo effect, which could reduce internal validity. Furthermore, responses could be affected by news related to the virus, vaccine side effects, and other related issues. Second, we could not provide evidence on the direction of vaccine hesitancy over time or the causal relationship between vaccine hesitancy and socio-economic variables because of the study’s cross-sectional nature. Third, as it was an internet survey, respondents were from a higher socio-economic status due primarily to unequal household income distribution in internet penetration rate [44]. Moreover, the age and gender distributions of this study differ to some extent from national statistics [45]. Fourth, several observations were excluded because of missing values for important variables, including financial literacy, household income, and household assets. Finally, despite our efforts to provide robust evidence on the variables associated with vaccine hesitancy of the younger generation, the results could be biased due to the unbalanced sample size. Nevertheless, the study provides detailed evidence of vaccine hesitancy among younger men and women in Japan. Future studies should use longitudinal data to confirm the direction of vaccine hesitancy and its causal relationship with socio-economic variables.

## Figures and Tables

**Table 1:** Variable definitions.

Variable	Definition
Dependent variables	
Vaccine hesitancy	Binary variable: 1 = Strongly disagree, disagree, neither agree nor disagree for the statement “Once the COVID-19 vaccination becomes available free of charge, I will take it soon.” and 0 = Otherwise
Explanatory variables	
Sex *	Binary variable: 1 = Male; 0 = Female
Age *	Continuous variable: Respondent’s age
Age squared *	Continuous variable: Respondent’s age squared
Spouse	Binary variable: 1 = Currently married; 0 = Otherwise
Children *	Binary variable: 1 = Have child/children; 0 = Otherwise
Living alone	Binary variable: 1 = Living alone; 0 = Otherwise
Living in central area *	Binary variable: 1 = Live in Kanto (around Tokyo metropolis) and Kinki (around Osaka metropolis); 0 = Otherwise
University degree *	Binary variable: 1 = Obtained university degree; 0 = Otherwise
Employed	Binary variable: 1 = Respondent employed; 0 = Otherwise
Household income	Continuous variable: Annual earned income before taxes/with bonuses of entire household in 2020 (unit: JPY)
Log of household income	Log (household income)
Household assets	Continuous variable: Balance of financial assets (savings, stocks, bonds, insurance) of entire household (unit: JPY)
Log of household assets	Log (household assets)
Financial literacy *	Continuous variable: Average score of correct answers from three financial literacy questions
Subjective health status	Ordinal variable. Based on the statement: “I am now healthy and was generally healthy in the last year.” 1 = It does not hold true at all for you; 2 = It is not so true for you; 3 = Neither true nor not true; 4 = It is rather true for you; 5 = It is particularly true for you
Anxiety about the future	Ordinal variable: Based on the statement: “I have anxieties about my ʻlife after I am 65 years oldʼ (for those who are already aged 65 or above: ʻlife in the futureʼ)” 1 = It does not hold true at all for you; 2 = It is not so true for you; 3 = Neither true nor not true; 4 = It is rather true for you; 5 = It is particularly true for you
Myopic view of the future	Ordinal variable: Based on the statement: “Since the future is uncertain, it is a waste to think about it.” 1 = Completely disagree; 2 = Disagree; 3 = Neither agree nor disagree; 4 = Agree; 5 = Completely agree
Level of risk preference	Continuous variable: Percentage score from the question “Usually when you go out, how high does the probability of rain have to be before you take an umbrella?”

Note: * variable from the 2020 dataset.

**Table 2:** Descriptive statistics.

Variable	Mean	SD	Min	Max
Vaccine hesitancy	0.53	0.50	0	1
Male	0.65	0.48	0	1
Age	50.32	13.83	21	86
Age squared	2723.05	1411.23	441	7396
Spouse	0.66	0.47	0	1
Children	0.57	0.49	0	1
Living alone	0.20	0.40	0	1
Living in central area	0.61	0.49	0	1
University degree	0.62	0.49	0	1
Employed	0.64	0.48	0	1
Household income	6,338,702	4,095,128	500,000	21,000,000
Log of household income	15.43	0.76	13.12	16.86
Household assets	19,800,000	29,100,000	1,250,000	125,000,000
Log of household assets	15.85	1.43	14.04	18.64
Financial literacy	0.65	0.36	0	1
Subjective health status	3.24	1.09	1	5
Anxiety about the future	3.71	1.14	1	5
Myopic view of the future	2.69	1.02	1	5
Level of risk preference	0.46	0.22	0	1
Observations	4253			

Note: SD: Standard Deviation.

**Table 3:** Vaccine hesitancy, stratified by gender and age.

Vaccine Hesitancy	Female				Male				Total
	Age < 35	Age 35–49	Age 50–64	Age ≥ 65	Age < 35	Age 35–49	Age 50–64	Age ≥ 65	
0	160	202	136	77	99	339	523	447	1983
	36.78%	36.20%	38.75%	61.11%	42.86%	40.17%	49.06%	69.63%	46.63%
1	275	356	215	49	132	505	543	195	2270
	63.22%	63.80%	61.25%	38.89%	57.14%	59.83%	50.94%	30.37%	53.37%
Total	435	558	351	126	231	844	1066	642	4253
	100%	100%	100%	100%	100%	100%	100%	100%	100%
Mean difference	F = 32.32 ***								

Note: *** *p* < 0.01.

**Table 4:** Results of probit regression analysis regarding younger and older females’ vaccine hesitancy.

Dependent Variable: Vaccine Hesitancy	Female: Age < 65	Female: Age ≥ 65
Age	0.0484 *	5.070 ***
	(0.0261)	(1.473)
Age squared	−0.000591 *	−0.0363 ***
	(0.000305)	(0.0104)
Spouse	−0.0775	−0.283
	(0.110)	(0.489)
Children	−0.182 **	−0.365
	(0.0858)	(0.417)
Living alone	−0.0891	−0.852
	(0.121)	(0.562)
Living in central area	−0.00801	0.152
	(0.0733)	(0.293)
University degree	−0.102	0.0648
	(0.0772)	(0.322)
Employed	−0.103	−0.200
	(0.0840)	(0.447)
Log of household income	−0.0767	−0.727 ***
	(0.0602)	(0.245)
Log of household assets	−0.00525	−0.0292
	(0.0330)	(0.114)
Financial literacy	−0.0696	−0.398
	(0.104)	(0.410)
Subjective health status	−0.156 ***	−0.162
	(0.0338)	(0.120)
Anxiety about the future	−0.0476	−0.0157
	(0.0341)	(0.121)
Myopic view of the future	0.0158	−0.123
	(0.0363)	(0.124)
Level of risk preference	0.155	0.238
	(0.174)	(0.692)
Constant	1.549	−163.8 ***
	(1.028)	(51.64)
Observations	1344	126
Log pseudolikelihood	−860	−67.34
Wald chi^2^	49.71	32.75
*p*-value	0.0000	0.00509
Pseudo R^2^	0.0294	0.200

Note: Robust standard errors are in parentheses; *** *p* < 0.01, ** *p* < 0.05, * *p* < 0.1.

**Table 5:** Results of probit regression analysis regarding younger females’ vaccine hesitancy.

Dependent Variable: Vaccine Hesitancy	Female: Age < 35	Female: Age 35–49	Female: Age 50–64
Age	0.201	0.774 ***	0.203
	(0.331)	(0.290)	(0.483)
Age squared	−0.00344	−0.00920 ***	−0.00211
	(0.00573)	(0.00345)	(0.00427)
Spouse	−0.398 **	0.0812	−0.0646
	(0.193)	(0.174)	(0.230)
Children	0.0283	−0.276 **	−0.179
	(0.165)	(0.134)	(0.167)
Living alone	−0.270	0.0549	−0.0593
	(0.206)	(0.193)	(0.264)
Living in central area	−0.0457	0.119	−0.0939
	(0.131)	(0.115)	(0.149)
University degree	−0.301 **	−0.00300	−0.00304
	(0.143)	(0.125)	(0.149)
Employed	−0.165	−0.215	0.214
	(0.153)	(0.133)	(0.170)
Log of household income	0.0138	−0.0425	−0.142
	(0.114)	(0.102)	(0.103)
Log of household assets	0.00407	−0.00865	0.00163
	(0.0706)	(0.0514)	(0.0592)
Financial literacy	0.0370	−0.143	−0.0495
	(0.190)	(0.165)	(0.209)
Subjective health status	−0.137 **	−0.209 ***	−0.137 *
	(0.0577)	(0.0538)	(0.0700)
Anxiety about the future	−0.106 *	−0.0620	−0.00776
	(0.0619)	(0.0559)	(0.0656)
Myopic view of the future	−0.0257	0.0241	0.0515
	(0.0634)	(0.0602)	(0.0712)
Level of risk preference	0.0735	0.284	0.0746
	(0.308)	(0.276)	(0.351)
Constant	−1.356	−14.03 **	−1.734
	(5.135)	(6.300)	(13.49)
Observations	435	558	351
Log pseudolikelihood	−275.2	−346.6	−222.8
Wald chi^2^	22.99	39.91	22.56
*p*-value	0.0844	0.000469	0.0939
Pseudo R^2^	0.0382	0.0510	0.0492

Note: Robust standard errors are in parentheses; *** *p* < 0.01, ** *p* < 0.05, * *p* < 0.1.

**Table 6:** Results of probit regression analysis regarding younger and older males’ vaccine hesitancy.

Dependent Variable: Vaccine Hesitancy	Male: Age < 65	Male: Age ≥ 65
Age	0.0813 ***	−0.0519
	(0.0240)	(0.306)
Age squared	−0.000963 ***	0.000275
	(0.000258)	(0.00211)
Spouse	−0.0908	0.119
	(0.0937)	(0.254)
Children	−0.0558	−0.624 ***
	(0.0760)	(0.167)
Living alone	−0.0148	0.0646
	(0.0916)	(0.278)
Living in central area	0.0318	−0.124
	(0.0588)	(0.113)
University degree	0.0548	−0.0595
	(0.0662)	(0.122)
Employed	0.0437	−0.0954
	(0.0988)	(0.128)
Log of household income	−0.103 **	0.199 *
	(0.0513)	(0.112)
Log of household assets	−0.0439 *	−0.102 **
	(0.0242)	(0.0463)
Financial literacy	−0.188 **	−0.243
	(0.0885)	(0.186)
Subjective health status	−0.136 ***	−0.0288
	(0.0266)	(0.0493)
Anxiety about the future	−0.104 ***	0.0586
	(0.0263)	(0.0559)
Myopic view of the future	0.0691 **	−0.0141
	(0.0282)	(0.0536)
Level of risk preference	0.0231	0.101
	(0.120)	(0.252)
Constant	1.592 *	1.035
	(0.882)	(11.28)
Observations	2141	642
Log pseudolikelihood	−1418	−376.6
Wald chi^2^	104.2	33.56
*p*-value	0	0.00393
Pseudo R^2^	0.0371	0.0447

Note: Robust standard errors are in parentheses; *** *p* < 0.01, ** *p* < 0.05, * *p* < 0.1.

**Table 7:** Results of probit regression analysis regarding younger males’ vaccine hesitancy.

Dependent Variable: Vaccine Hesitancy	Male: Age < 35	Male: Age 35–49	Male: Age 50–64
Age	0.128	−0.338	0.0409
	(0.460)	(0.239)	(0.269)
Age squared	−0.00114	0.00403	−0.000617
	(0.00793)	(0.00280)	(0.00237)
Spouse	−0.385	−0.119	−0.0329
	(0.279)	(0.156)	(0.135)
Children	0.161	0.0824	−0.187 *
	(0.269)	(0.129)	(0.105)
Living alone	−0.107	0.0554	−0.0655
	(0.272)	(0.141)	(0.143)
Living in central area	0.0838	0.0795	−0.0308
	(0.185)	(0.0942)	(0.0844)
University degree	−0.429 *	0.0633	0.118
	(0.244)	(0.107)	(0.0923)
Employed	−0.483	0.120	0.107
	(0.319)	(0.192)	(0.127)
Log of household income	0.211	−0.160 *	−0.120 *
	(0.212)	(0.0895)	(0.0684)
Log of household assets	−0.188 *	−0.0116	−0.0471
	(0.103)	(0.0399)	(0.0332)
Financial literacy	−0.604 **	−0.255 *	−0.0403
	(0.259)	(0.138)	(0.133)
Subjective health status	−0.0184	−0.106 **	−0.177 ***
	(0.0877)	(0.0434)	(0.0373)
Anxiety about the future	−0.344 ***	−0.126 ***	−0.0624 *
	(0.0920)	(0.0440)	(0.0370)
Myopic view of the future	0.134	0.00861	0.0927 **
	(0.0871)	(0.0457)	(0.0403)
Level of risk preference	0.424	−0.334 *	0.158
	(0.442)	(0.201)	(0.162)
Constant	−0.948	10.87 **	2.828
	(7.300)	(5.205)	(7.602)
Observations	231	844	1066
Log pseudolikelihood	−139.2	−551	−705.1
Wald chi^2^	37.31	33.86	64.41
*p*-value	0.00114	0.00356	4.34e–08
Pseudo R^2^	0.118	0.0309	0.0455

Note: Robust standard errors are in parentheses; *** *p* < 0.01, ** *p* < 0.05, * *p* < 0.

**Table 8:** Results of multinomial probit regression analysis regarding younger females’ vaccine hesitancy.

Dependent Variable: Vaccine Hesitancy	Female: Age < 35	Female: Age 35–49	Female: Age 50–64
Age	1.937 ***	2.834 ***	3.492 ***
	(0.300)	(0.197)	(0.404)
Age squared	−0.035 ***	−0.034 ***	−0.031 ***
	(0.005)	(0.002)	(0.004)
Spouse	−0.572 ***	−0.037	0.200
	(0.255)	(0.232)	(0.273)
Children	0.062	−0.319 *	−0.372 *
	(0.210)	(0.171)	(0.209)
Living alone	−0.254	0.024	−0.004
	(0.264)	(0.247)	(0.307)
Living in central area	−0.152	0.182	−0.124
	(0.168)	(0.149)	(0.186)
University degree	−0.406 **	−0.065	0.098
	(0.186)	(0.159)	(0.189)
Employed	−0.269	−0.233	0.141
	(0.197)	(0.173)	(0.214)
Log of household income	−0.006	0.004	−0.288 **
	(0.145)	(0.122)	(0.140)
Log of household assets	0.033	−0.022	−0.033
	(0.086)	(0.065)	(0.076)
Financial literacy	−0.051	−0.167	0.094
	(0.251)	(0.214)	(0.263)
Subjective health status	−0.209 ***	−0.279 ***	−0.189 **
	(0.076)	(0.069)	(0.085)
Anxiety about the future	−0.134 *	−0.115	−0.027
	(0.079)	(0.071)	(0.080)
Myopic view of the future	−0.061	0.017	0.126
	(0.084)	(0.077)	(0.090)
Level of risk preference	0.122	0.309	−0.024
	(0.401)	(0.351)	(0.451)
Constant	24.600 ***	−56.740 ***	−92.480 ***
	(5.023)	(4.387)	(11.060)
Observations	1344	1344	1344
Log pseudolikelihood	−920.020	−920.020	−920.020
Wald chi^2^	560.2	560.2	560.2
*p*-value	0.00	0.00	0.00

Notes: Base outcome: vaccine non-hesitancy among younger females. Robust standard errors are in parentheses; *** *p* < 0.01, ** *p* < 0.05, * *p* < 0.1.

**Table 9:** Results of multinomial probit regression analysis regarding younger males’ vaccine hesitancy.

Dependent Variable: Vaccine Hesitancy	Male: Age < 35	Male: Age 35–49	Male: Age 50–64
Age	2.473 ***	2.275 ***	2.527 ***
	(0.418)	(0.140)	(0.212)
Age squared	−0.0433 ***	−0.027	−0.022 ***
	(0.007)	(0.002)	(0.002)
Spouse	−0.599	−0.260	0.069
	(0.340)	(0.190)	(0.175)
Children	0.366	0.153	−0.270 *
	(0.309)	(0.158)	(0.138)
Living alone	−0.117	0.048	0.005
	(0.320)	(0.175)	(0.178)
Living in central area	0.152	0.095	−0.038
	(0.221)	(0.117)	(0.110)
University degree	−0.466 *	0.102	0.144
	(0.275)	(0.133)	(0.121)
Employed	−0.518	−0.046	0.088
	(0.370)	(0.219)	(0.173)
Log of household income	0.226	−0.157	−0.179 *
	(0.223)	(0.105)	(0.091)
Log of household assets	−0.170	0.039	−0.063
	(0.108)	(0.049)	(0.044)
Financial literacy	−0.696 **	−0.371 ***	−0.149
	(0.314)	(0.170)	(0.171)
Subjective health status	0.049	−0.156 ***	−0.250 ***
	(0.112)	(0.054)	(0.048)
Anxiety about the future	−0.361 ***	−0.193 ***	−0.095 **
	(0.101)	(0.053)	(0.048)
Myopic view of the future	0.136	0.072	0.117 **
	(0.103)	(0.056)	(0.053)
Level of risk preference	0.217	−0.319	0.147
	(0.492)	(0.248)	(0.210)
Constant	−33.120	42.670 ***	−66.540 ***
	(7.020)	(3.249)	(5.756)
Observations	2141	2141	2141
Log pseudolikelihood	−1551	−1551	−1551
Wald chi^2^	872.3	872.3	872.3
*p*-value	0.00	0.00	0.00
Pseudo R^2^			

Notes: Base outcome: vaccine non-hesitancy among younger males. Robust standard errors are in parentheses; *** *p* < 0.01, ** *p* < 0.05, * *p* < 0.

## Data Availability

Data is available on request.
